# “… I carry their stories home …”: experiences of nurses and midwives caring for perinatal adolescent mothers in primary health care settings in Rwanda

**DOI:** 10.1186/s12912-024-02247-7

**Published:** 2024-09-02

**Authors:** Aimable Nkurunziza, Victoria L. Smye, Kimberley T. Jackson, C. Nadine Wathen, David F. Cechetto, Panagiota Tryphonopoulos, Darius Gishoma, Alice Muhayimana

**Affiliations:** 1 College of Medicine and Health Sciences, School of Nursing and Midwifery, University of Rwanda, Kigali, Rwanda; 2 Faculty of Health Sciences, Arthur Labatt Family School of Nursing, Western University, London, ON Canada; 3 Lawrence Bloomberg Faculty of Nursing, University of Toronto, Toronto, ON Canada; 4 School of Nursing, Nipissing University, North Bay, Canada; 5 Schulich School of Medicine & Dentistry, Western University, London, ON Canada; 6 Rwanda Biomedical Center, Mental Health Division, Ministry of Health, Kigali, Rwanda; 7 School of Therapeutic Sciences, Department of Nursing Education, University of Wiwatersrand, Johannesburg, South Africa

**Keywords:** Adolescent mother, Nurses, Midwives, Perinatal, Primary care settings, Trauma- and violence- informed care, Structural violence

## Abstract

**Introduction:**

Adolescent mothers require trauma- and violence-informed care during the perinatal period due to trauma histories and ongoing violence as a result of pregnancy. Nurses and midwives play a critical role in caring for adolescent mothers in primary healthcare settings in Rwanda in the perinatal period.

**Purpose:**

To explore the experiences of nurses and midwives working with adolescent mothers in selected primary healthcare settings in Rwanda to inform the delivery of trauma- and violence- informed care.

**Methods:**

This study utilized an interpretive description qualitative approach and was conducted in eight primary healthcare settings in Rwanda. Twelve nurses and midwives working in perinatal services and four heads of health centers participated in in-depth individual interviews. Data were analyzed thematically.

**Results:**

The analysis revealed four main themes and 11 (sub-themes): (a) relational practice (being creative and flexible, “lending them our ears”); (b) individual challenges of providing care to adolescent mothers (lack of knowledge to provide care related to gender-based violence, and gendered experience); (c) factors contributing to workarounds (inflexible guidelines, lack of protocol and procedures, lack of nurses’ and midwives’ in service training, and the physical structure of the perinatal environment); and (d) vicarious trauma (living the feelings, “I carry their stories home,” and hypervigilance in parenting).

**Conclusion:**

Nurses and midwives find caring for adolescent mothers challenging due to their unique needs. These needs require them to be creative, adaptable, and attentive listeners to better understand their challenges. These practitioners face difficulties such as insufficient specific knowledge related to, for example, gender-based violence, inflexible guidelines, and a lack of protocols and training. Additionally, in the perinatal environment attention to the needs of practitioners in those settings is often lacking, and many nurses and midwives report experiencing vicarious trauma. Consequently, there is a pressing need for guidelines and protocols specifically tailored for the care of adolescent mothers. Ongoing trauma- and violence- informed care training and professional education should be provided to enhance the ability of nurses and midwives to care for adolescent mothers and prevent re-traumatization and mitigate vicarious trauma effectively.

**Supplementary Information:**

The online version contains supplementary material available at 10.1186/s12912-024-02247-7.

## Background

The teenage pregnancy rate is alarming in low-income countries (LMICs), with approximately 21 million adolescent girls aged between 15 and 19 becoming pregnant [1]. In comparison to other LMICs, Sub-Saharan Africa (SSA) has the highest prevalence [2–6] and the East African Community (EAC) accounts for nearly a quarter [7]. The teen pregnancy rate in Rwanda, one of the EAC countries, is 5% among girls aged 15–19 (National Institute of Statistics of Rwanda [NISR] et al., 2020) and 75% of these pregnancies are related to sexual violence or coercion [8].

Adolescent pregnancy can be traumatic and is associated with many traumatic events [9, 10] resulting in poor mental health outcomes such as post-traumatic stress disorder (PTSD), depression, substance abuse, and suicide [9, 11, 12]. Failure to take into account these unique challenges when caring for adolescent mothers can re-traumatize them [13]. Thus, systems of care need to adopt trauma- and violence- informed approaches to adequately address the needs of adolescent mothers. According to Wathen and Varcoe [14], “Trauma can also result from what does not happen, for example, when systems fail to recognize and intervene in gender-based violence and its related causes and consequences” (p. 3). A plethora of research has shown the importance of incorporating trauma-informed approaches in perinatal services [15–17].

Perinatal nurses and midwives have different experiences when working with adolescent mothers. For example, in Canada, perinatal nurses reported that they deliver a positive experience and ensure that adolescent mothers and their babies feel safe [18]. They achieve this through non-judgmental approach, developing therapeutic relationship, and adjusting care according to the adolescents’ needs [18]. However, a study conducted in Jamaica conducted with midwives practicing in one selected hospital revealed that over a half of the midwives had negative or neutral attitudes towards adolescent mothers [19].

Most adolescent mothers from Rwandan families are of low socio-economic status and utilize primary healthcare settings (health posts and health centers) since they cannot afford the costs of public hospitals and private clinics [20]. These health centers are run by nurses and midwives [21] and manage 85% of healthcare needs of the population [22]. Notably, it has been demonstrated that only 14% of nurses and midwives working in antenatal care in perinatal services in Rwanda are adequately prepared to care for a client with a history of sexual violence [23], which shows the importance of exploring the experiences of nurses and midwives when caring for adolescent mothers and the provision of trauma- and violence-informed care (TVIC) approach.

TVIC focuses on creating safety for people by understanding how trauma impacts health and behaviour as well as the intersecting impacts of systemic and interpersonal violence and structural inequities on people’s lives. It emphasizes the traumatic effects of both historical and ongoing violence, drawing attention to people’s past and current experiences of violence, stigma, and discrimination, arguing that the effects of violence and stigma are based on their psychological state and social circumstances, as well as permeating society and culture [13]. Healthcare provision that is trauma- and violence-informed is underpinned by the following principles. The need to: (1) understand trauma and violence and its impacts on someone’s life and behavior; (2) create emotionally and physically safe environments for all clients and service providers; (3) foster opportunities for choice, collaboration, and connection; and (4) use a strengths-based approach and capacity building to support clients [13].

Trauma and violence are prevalent in many forms, including interpersonal violence and structural violence, making it safe to assume that anyone seeking help has been impacted by either or both [13]. For making policies and practices safe, TVIC emphasizes that there needs to be an understanding of trauma and ongoing violence among individuals and communities [24] and the intersection of interpersonal and structural forms of violence and their impact on health and well-being [25]. Despite the potential advantages TVIC can offer when caring for the unique needs of perinatal adolescent mothers, there is a paucity of literature on how nurses and midwives care for adolescent mothers in Rwandan primary healthcare settings. Thus, this study explored the experiences of nurses and midwives working with adolescent mothers in the selected healthcare centers in Rwanda to inform the delivery of TVIC.

## Methods

### Study design

This study utilized an interpretive description (ID) methodology which is a qualitative approach developed by nursing scholars to generate knowledge around complex clinical issues in nursing [26]. ID assists nurse researchers to “build methods that are grounded in our own epistemological foundations, adhere to the systematic reasoning of our discipline, and yield legitimate knowledge for our practice” [26].

### Study setting

This study was conducted in eight selected primary healthcare settings in Rwanda because of the high adolescent pregnancy rate in their catchment areas [27]. These primary healthcare settings were selected based on their accessibility. Primary healthcare settings handle 85% of the population needs [28] and as of 2022, occupy 56% of all public health facility beds [29]. In these settings, nurses and midwives are responsible of all clinical decisions [30, 31]. Primary healthcare settings in Rwanda offer a comprehensive range of services [30]. They provide promotional activities like health education, psychosocial support, nutritional programs, community engagement, home visits, and hygiene and sanitation initiatives. Preventive services include premarital consultations, antenatal and postpartum care, newborn care, family planning, school health programs, epidemiologic surveillance and outreach activities such as vaccinations. Curative services encompass medical consultations, management of non-communicable and communicable diseases, normal deliveries, minor surgical procedures, and basic laboratory testing. These diverse services ensure a holistic approach to healthcare. This integration helps address the majority of healthcare needs at the community level. Each healthcare setting has approximately 30 staff [32].

### Study participant recruitment

In ID, it is essential to select the study participants who are willing to share their experiences [33]. Therefore, purposive sampling was used to select nurses and midwives and four key informants (KIs). Managers of health centers oversee health centers’ activities. We recruited the study participants based on the following criteria. For nurses and midwives: they (a) worked in perinatal services (Prevent Mother To Child Transmission [PMTCT], Antenatal Care [ANC], and Maternity); (b) provided care at least one year in perinatal services; and (c) were willing to share their experiences of caring adolescent mothers in perinatal services. For KIs: they (a) were the Head of a health center; (b) had been in their role for at least one-year; and b) were willing to share their firsthand knowledge in overseeing perinatal services activities.

### Data collection procedures

Ethical approval was obtained from Western University Research Ethics Board (HSREB, project ID: 119846) and the University of Rwanda-Institutional Review board (No 330/CMHSIRB/2021). Following these approvals, the researcher sought permission from the study sites, which authorized the research team to access the potential study participants in staff meetings. Participating health centers advertised the study and those interested in participating contacted the researcher through their shared email or telephone contact information. Those who met the inclusion criteria were provided additional information about the study, and informed consent was obtained by those agreeing to participate. An in-depth, one-to-one interview was conducted with the participant and two research assistants present. In four primary healthcare settings, a research team member (AN) secured two quiet staff offices at the selected institution. In the other four settings, AN worked with the head of the health center to identify an appropriate room. The interviews occurred at a time that was convenient in consideration of the study participants’ work schedules.

The interview guides (Supplementary 1&2) were developed by the research team based on the literature [18, 34–39] and translated back by a certified translator fluent in both English and Kinyarwanda and is familiar with the local context. The research team members (AN & DG), who are fluent in both English and Kinyarwanda, approved the interview guides. The interview guides included open-ended, invitational/probing questions to engage the participants in discussion. The interview was conducted in Kinyarwanda, which is the mother tongue of participants. Two RAs, skilled in qualitative research and fluent in both English and Kinyarwanda, assisted with data collection.

For recruitment, sampling, and data collection, we used the ID principle of ‘disciplinary logic.’ This principle includes identifying the needed knowledge, selecting the best methods to closely examine the subject of interest, and conducting inquiries in line with ethical and research guidelines [40]. To determine the sample size, we were guided by information power, which is a more logical and defensible alternative to data saturation [41] and suggests that fewer participants are needed if the sample contains more relevant information [42]. In this study, we recruited experienced participants who were willing to share their experiences of caring for adolescent mothers in perinatal services and employed experienced RAs. As a result, 12 nurses and midwives and four KIs provided rich data. Interviews were audio-recorded and field notes were taken during and after the interviews to record the participants’ reactions and non-verbal communication. The interviews ranged between 36 and 52 min. After the interview, each study participant received a $10 CDN honorarium (8,000 Rwandan francs) to compensate for their time, mobile phone airtime and transportation.

### Data analysis

ID supports concurrent data collection and analysis because one iteratively influences the other. In ID, there is a social construction of reality and researchers engage with data as soon as they enter the field [40]. Data were transcribed verbatim, and translated into English by the researcher (AN). We used Dedoose software to sort and organize data. We followed Braun and Clarke’s framework for thematic analysis and included the following steps in keeping with their recommendations [43]: familiarizing with the data, generating initial codes, searching for themes, reviewing themes, defining and naming themes, and producing the report. After coding the two research team members (AN and VS) agreed on the codes. Coders held regular meetings to discuss and resolve differences in coding to reach a consensus [44]. The coded data were then analyzed to identify patterns and themes. The entire research team discussed and interpreted themes and patterns. The construction of these themes remained provisional as we continued our analysis in our meetings. This approach allowed us to modify or restructure them as they evolved, and they could be redefined at higher or lower levels of abstraction [45].

### Rigor

To ensure the integrity and rigor of our findings, we carefully followed the four evaluative criteria of ID studies outlined by Thorne [40]: epistemological integrity, representative credibility, analytic logic, and interpretive authority. To meet these criteria, we employed the following strategies: acknowledging the epistemological standpoint consistent with the research question, identifying the interpretive strategies that logically follow, reflexive journaling, methodological triangulation, maintaining an audit trail, analyzing field notes, and member checking. Considering the larger disciplinary, historical, and social contexts in which our research was conducted, we also took into account additional criteria beyond the four evaluative criteria. These include moral defensibility, disciplinary relevance, pragmatic obligation, contextual awareness, and probable truth [40].

## Findings

Twelve perinatal staff (five midwives and seven nurses) who work in perinatal services were recruited. All but one was over 30 years old and all were advanced diploma nurses or midwives; half of the participants had been employed in perinatal services > 10 years (Table 1).

**Table 1:** Socio-demographic characteristics of perinatal nurses and midwives

No.	Pseudonym	Profession	Age	Gender	Level of education	Working experience
1.	MA	Midwifery	43	Female	A1	17 years
2.	MB	Midwifery	39	Female	A1	9 years
3.	MC	Midwifery	43	Female	A1	7 years
4.	MD	Midwifery	25	Male	A1	2years
5.	ME	Midwifery	33	Female	A1	8 years
6.	NA	Nursing	41	Female	A2	10 years
7.	NB	Nursing	37	Female	A1	13 years
8.	NC	Nursing	38	Male	A1	8 years
9.	ND	Nursing	39	Female	A2	12 years
10.	NE	Nursing	38	Female	A2	10 years
11.	NF	Nursing	41	Female	A1	6 years
12.	NG	Nursing	36	Female	A1	16 years

Four heads of health centers participated in this study and two of them had > 5 years of experience in this role (Table 2).

**Table 2:** Socio-demographic characteristics of key informants

No	Title	Age	Gender	Level of education	Working experience
KI1	Head of health center	45	Male	A1	15 years
KI2	Head of health center	48	Female	A0	17 years
KI3	Head of health center	42	Male	A1	1 year
KI4	Head of health center	36	Male	A1	1 year

### Themes

Four main themes with their (sub-themes) were identified during the thematic analysis: (a) relational practice (being creative and flexible, “lending them our ears”); (b) individual challenges of providing care to adolescent mothers (lack of knowledge to provide care related to gender-based violence, and gendered experience); (c) factors contributing to workarounds (inflexible guidelines, lack of protocol and procedures, lack of nurses’ and midwives’ in service training, and physical structure of the perinatal environment); and (d) vicarious trauma (living the feelings, “I carry their stories home,” and hypervigilance in parenting) (Table 3).

**Table 3:** Selected codes, sub-themes, and themes of the experiences of nurses and midwives caring for perinatal adolescents

Themes	Sub-themes	Selected codes
Relational practice	Being creative and flexible	Juggling multiple skills
		Time consuming
		Interdisciplinary collaboration
	“Lending them our ears”	Empathy
		Forming relationships
		Negotiating consent
		Non-judgmental care
Individual challenges of providing care to adolescent mothers	Lack of knowledge to provide care related to gender-based violence	GBV knowledge and skills
		Academic background
		Need of refresher training
	Gendered experience: Being a male nurse caring for an adolescent mother	Refusal of care
		Female provider involvement
Factors contributing to workarounds	Inflexible guidelines	Presenting partners
		Community health insurance
	Lack of protocols and procedures	Insufficient information
		Charts
		Guidelines
	Lack of nurses’ and midwives’ in service training	Common sense
		Unnecessary referrals
		Waiting times
	Physical structure of the perinatal environment	Common areas
		Medical supplies
		Confidentiality
Vicarious trauma	Living the feelings	Emotional drainage
		Sadness
		Shocking
	“I carry their stories home”	Depression
		Projection
	Hypervigilance in parenting	Loss of trust in males
		Dating relationships
		Health education

#### Theme one: relational practice

When connecting with adolescent mothers in perinatal services, all nurses and midwives described caring for an adolescent mother as challenging. However, they also added that they try their best to care for the adolescent mothers by being creative and flexible and listening to the mother with empathy.

- ***Being creative and flexible***

Even though much of the care provided in perinatal services is routine, i.e., it is provided to every woman in perinatal services, nurses and midwives reported that there are additional demands which can be time-consuming when caring for adolescent mothers. For example, a few nurses and midwives reported that caring for an adolescent mother requires juggling multiple skills. One midwife said,

*“It is challenging and time consuming. There can be a time when you can spend three hours without getting her consent for a single procedure such as a vaginal examination because she is mostly in pain.”* (ME).

Another nurse described this experience as juggling multiple tasks. As they said,

*“Ah*,* it’s tough to care for these adolescents; generally*,* it’s difficult. You have to deal with many things at once….*

*There is one [adolescent mother] that I received when examining her*,* she squeezed her legs together*,* refused completely*,* and I failed to examine her. I even approached the person who had brought her to help me*,* but it did not work. I told myself that instead of having a certain [negative] incident either for the mom or the baby*,* I’d rather transfer her to the hospital.*”(ND).

Health center managers also noted that caring for adolescent mothers can take considerable time; in response they sometimes get involved helping nurses and midwives on duty. As one health center manager said,

*“In morning reports*,* adolescent mothers are reported to give nurses/midwives a hard time. I have often been called during the day or at night to assist them with situations when a nurse/midwife spent long time negotiating with an adolescent without any positive results.”* (KI3).

Nurses and midwives noted differences between caring for adolescent mothers and adult women which they tried to accommodate. Here, the nurse believed that the potential safety risk for the mother and baby should be addressed by transferring the adolescent mother to the district level for care. However, this would have added additional costs to the adolescent mother and the family, given the transportation payment and the costs associated with district hospital services.

One nurse stated that sometimes receiving an adolescent mother is challenging due to her complex needs. They added that when they receive the adolescents in ANC visits, they often have to counsel them for other issues such as abortion and suicidal ideation. A few of nurses and midwives ensure that adolescent mothers feel safe and are involved in care as explained,

*“I counsel her to make her feel that you are together. I explain everything that giving birth is a process that requires her to put her efforts.”* (NG).

Another midwife provides an example of being flexible when caring for an adolescent mother in the following:

*“From the first time of receiving her up to discharge or referral*,* even after discharge*,* you have to expect that whatever you will do is quite different from other mothers. [For example] you have to deal with delayed consent before the procedures*,* and provide more explanations. So*,* you have to be patient!”* (MD).

Some key informants (KIs) said they were responsible for reminding nurses and midwives about the importance of ensuring that adolescent mothers are safe and involved in their care. For example, as one KI noted,

*“Well*,* of course*,* we always encourage our staff to consider adolescent mothers as special and to provide them with additional information to understand what is happening to them during their care.”* (KI**1**).

Advocacy is another concept that emerged from the study participants’ narratives. Given the vulnerability of this patient population, e.g., financial instability, being judged and stigmatized and even abused in their communities and because there are no particular care guidelines related to the care of adolescent mother in perinatal services, some nurses and midwives felt they needed to advocate for proper care management and referrals. As one nurse notes in the following,

*“I approach the head of the health center to find out how we can register the adolescent mother under the 1st category of community-based health insurance. Then the cost is paid by the district office.”* (NC).

In a similar vein, another midwife speaks to advocacy in the following:

“*There is a project which takes care of those adolescent mothers. We contact them and connect her with that project. They help her.”* (MC).

In response to advocacy by nurses and midwives, some KIs helped adolescent mothers in these situations. As one KI said,

*“I am always contacted by them [nurses and midwives] to inform me that they [adolescent mothers] cannot pay. We discuss how we can help.”* (KI**3**).

Financial instability and the inability to pay for community health insurance is one of the biggest barriers to access perinatal services for adolescent mothers.

A few nurses and midwives reported engaging in interdisciplinary collaboration as a pillar in the proper management of adolescent mothers in perinatal services. They described caring for adolescent mothers as primarily the responsibility of nurses and midwives, however, they also pointed to the roles and importance of a nutritionist, community health officer, social worker, and a mental health nurse as part of perinatal care services. As one midwife said,

*“In some cases*,* we decide that a nutritionist*,* a social worker and a community health officer work together to take care of vulnerable adolescents since they often come from poor families or are rejected.”* (MB).

Another midwife added that mental health problems are more prevalent in perinatal services, and sometimes perinatal nurses’ and midwives’ skills in counselling are limited. For example, a midwife shared the experience of a 17-year-old adolescent mother who had been rejected by the father of her baby, resulting in mental health issues which required interdisciplinary collaboration. Because of the severity of her symptoms, the family brought her to the health center for counselling. This midwife added that it went beyond their capacity as nurses and midwives to provide this kind of care because their skills are limited. A midwife explained,

*“There is a nurse in charge of mental health. When you realize that you may not be able to make good counselling sessions*,* you ask her for assistance. She works every day until Saturday.”* (MC).

Even though these settings are in rural areas, having a mental health nurse working until Saturday was one of the strengths of the interdisciplinary team because they would serve adolescent mothers whenever they needed care. Understandably, working in an interdisciplinary team helped these nurses provide quality care to these adolescent mothers. In addition, it prevented adolescent mothers and their families from paying additional costs related to transfer to district hospitals for further management of mental health problems.

(b) ***“Lending them our ears”***.

The nurses and midwives interviewed discussed the need to ensure that adolescent mothers receive non-judgmental care. A few of them noted that active listening is a top priority. For example, as one nurse reported,

*“I do not judge or blame them [adolescent mothers]. They had been and are still being blamed so much in the community and families. As a nurse*,* I must show her love and the difference and listen to them with sympathy.*” (NE) A midwife added, *“Hmm… one thing I found that is important is listening to them…. well*,* lending them our ears.”* (MB).

, The quotes above exemplify how these nurses and midwives recognize the impact of family and community stigma on an adolescent mother’s life; they wanted to ensure the adolescent mothers felt full acceptance instead of judgement – listening was an essential element of that process.

Even though it is challenging to care for adolescent mothers in perinatal services, some nurses and midwives reported that they overcome these challenges by fostering connection and trust with the mother. A midwife shared how creating a positive relationship helped her care for an adolescent mother with several problems. She assisted a 15-year-old girl in the ANC who was impregnated by a local leader after promising her some school materials. According to the adolescent, this man beat and insulted her; she was traumatized and in response requested an abortion. However, the midwife spent time with the adolescent, explained the issue and the consequences that could follow so that she could make an informed decision. The adolescent mother agreed to give birth, and now she is happy with her baby and always comes to see that midwife. At delivery, the adolescent mother was referred to the district hospital for management. However, the relationship between the midwife and the adolescent mother continued to grow, as noted in the following quote:

*“I found that the conversation we had made her come back looking for me*,* and now she keeps coming to see and tell me ‘see your baby.’ Now*,* her baby is my baby too… These words keep motivating me in my daily practice to form a connection and build a positive relationship with adolescent mothers.” (*MB).

In the situation above, the midwife demonstrated that listening to this young mother and developing a positive relationship resulted in long term positive outcomes such as adolescent mother’s informed decision to go forward with the pregnancy and the care of her baby. The midwife considered keeping the pregnancy the only positive outcome, while abortion was considered a possible option by the adolescent mother.

#### Theme two: individual challenges of providing care to adolescent mothers

From the participants’ narratives, it was revealed that the majority of nurses and midwives perceived some challenges when connecting with adolescent mothers. These challenges included a lack of knowledge and skills, and a gendered experience.

- ***Lack of knowledge to provide care related to gender-based violence (GBV)***

Some nurses and midwives reported challenges which hindered the quality care of care provision with adolescent mothers, including gaps in knowledge and skills specific to GBV against adolescent mothers’ care. For example, a midwife in this study reported the following:

*“I have no special knowledge of caring for an adolescent with a history of violence. I have to help her in every way possible*,* and I refer her to Isange One Stop Center*,* which would help her with anything. The only other knowledge I have to help these adolescent mothers is that if she does not accept giving birth*,* they can have an abortion. If she is a GBV case*,* she has the right to abort. Yeah*,* that’s it. I believe that my knowledge is not sufficient. I need sufficient knowledge because what I do is what I can help anyone else who is not an adolescent mother. For sure*,* I need to upgrade my knowledge and skills to take care of GBV cases.”* (MB).

In this case, the midwife did not feel she had the ability to provide care adequately related to gender-based violence (GBV). The Isange One Stop Center supports the national efforts of the Ministries of Health, National Police and Justice to combat GBV. As the name Isange Centre implies, “feel free/feel welcome,” the center communicates a message of security and openness to survivors. A lack of specific knowledge was also reported by another participant,

*“I talk to them [adolescent mothers] as a parent but [have] no other specific knowledge””* (ND).

A midwife notes her need in the following,

*“Personally*,* knowledge isn’t a problem. What is needed in terms of knowledge is to ‘refresh*,*’ to do a refresher training on how to take care of this special population.”* (MD).

KIs also reported nurses’ and midwives’ limited knowledge to care for adolescent mothers. Nurses and midwives are cognizant that adolescent mothers need special care because of their unique needs. Inconsistencies in care were related to the years of experience, time since finishing school, and/or academic background (i.e., midwifery vs. nursing). For example, the nurse participants we interviewed who recently graduated reported utilizing knowledge from school, while those more experienced providers found recalling information more challenging.

(b) ***Gendered experience***

All male nurses and midwives reported that caring for adolescent mothers is sometimes difficult. Several cases were described where adolescent mothers refused a male nurse or told male nurses/midwives not to touch them because they were male. As one male nurse said,

*“Because I am a male health care provider*,* sometimes*,* they fear me. I have met with some [adolescent mothers] who refused me to examine them because I am a male provider. One told my female colleague that men are not serious. If she does not want*,* you don’t have to force*,* better to call female colleagues to help.”* (NC).

In this similar context, the male midwife MD shared the story of a 15-year-old girl who came to give birth. While the girl was experiencing significant labor pain, he had to conduct a vaginal exam to ascertain the extent of dilation. He explained the process and the need to examine her, yet she refused. Upon further assessment, he realized that the girl was refusing to be examined because he was male so he called upon a female nurse/midwife who explained everything to the adolescent mother. All these nurses and midwives recognized that forcing the adolescent mothers could result in negative outcomes. Thus, they have been able to involve female providers to ensure the safety and comfort of their clients.

#### Theme three: factors contributing to workarounds

Most nurses and midwives reported the use of workarounds in perinatal care when caring for adolescent mothers as related to stringent guidelines, a lack of protocols and procedures and lack of nurses’ and midwives’ education and training and the unwelcoming nature of the perinatal care environment.

- ***Inflexible guidelines***

A few nurses and midwives reported that when caring for adolescent mothers in perinatal services, they follow guidelines that are not flexible; that is, they do not accommodate the needs of the adolescent mother. For example, adolescent mothers are mandated to bring their husbands or partners on their initial visit. This requirement can re-traumatize them because they often do not have or cannot locate their partners. According to Article 194 of the Rwandan Penal Code, anyone living with a child as a husband or wife faces life imprisonment. Therefore, it’s almost impossible to find these men/boys. One midwife noted,

*“For their husbands*,* you cannot find any because they might be jailed because it is criminalized”* (MB).

Most of the nurses and midwives have said that they do not see why they ask that and believe that this is something which can be changed for the sake of helping adolescent mothers. As one midwife said,

*“Everyone you tell to bring a partner becomes unhappy and sometimes may cry.”* (MB).

When asked why they ask adolescent mothers to bring their partners while they are sure that they cannot find them, she replied,

*“That is how guidelines are structured. You have to follow them and tick in the register that you have done that.”* (MB).

A nurse shares their response to the guideline as follows,

*“If she doesn’t find a partner or I realize that if she goes back*,* she will not come back*,* I help her regardless of the rules. However*,* it’s not accepted. She should bring an authorization copy from the local authorities.”* (NF).

However, the head of the health center KI3 said,

*“So*,* here*,* we can’t ask them [adolescent mothers] to bring their husbands.”* KI**3**.

These inconsistencies may be related to the type of services an adolescent mother will receive, either emergency or non-emergency. As a nurse noted,

*“Sometimes they [adolescent mothers] don’t get services directly*,* especially if they do not have insurance*,* but the health center’s manager helps us to resolve these issues.”* (NG).

and when asked why an adolescent mother does not get the services right away, they said,

*“It delays because we need to inform the health center head that adolescent mothers do not have health insurance. So*,* it may take some time to sort it* out.*”* (NG).

In the cases noted above, nurses and midwives knew the guidelines were inflexible and bypassed them to help the adolescent mothers. Nurses and midwives recognized the negative effects of sending adolescent mothers back to community health workers or executives of the community cell to bring a confirmation paper that provided a reason why they could not attend clinic with their husbands. Thus, they chose to help them get services instead of sending them back to the community.

(b) ***Lack of protocols and procedures specific to adolescent mothers***

In this study, nurses and midwives noted the tensions and disjunctures created between the guidelines re: perinatal care and what adolescent mothers needed. They expressed concern that the existing guidelines contain insufficient information, often limited to just a single paragraph. They emphasize that caring for an adolescent mother involves addressing the needs of a whole person who often requires more care than other mothers. This inadequacy in guidelines makes it challenging to provide appropriate care, leaving nurses and midwives uncertain about the effectiveness of their support. For example, as one nurse said,

*“We do not have special guidelines or protocols for adolescent mothers. In the guidelines*,* some information about adolescent mothers is insufficient. You will find that it’s only one paragraph*,* but when you take care of an adolescent mother*,* it’s a whole person who even needs more care than other mothers. It’s challenging and sometimes you are sure that*,* even though you helped her [adolescent mother]*,* you did not do it appropriately.”* (NA).

The health center managers underscore the absence of the necessary and written guidelines for caring for adolescent mothers. They point out the need for detailed, step-by-step protocols that guide nurses and midwives on how to receive and communicate with adolescent mothers. They also suggest that such instructions should be displayed in offices to serve as references during consultations so staff can access them easily. For example, as one head of the health center noted,

*“No charts are available. We do not have the written instructions to show us how to care for an adolescent mother from point a*,* b*,* c…. How to receive and talk to them* …*Yet they should be hung somewhere in the offices to be used as references during consultations*,* as protocols and guidelines so that it is well-known what to do.”* (KI**4**).

(c) ***Lack of on job training for nurses and midwives***

Some nurses and midwives highlighted a lack of special training as challenges they face in their daily practices in perinatal services when connecting with adolescent mothers. They emphasized the need to update their knowledge since they still rely on school-level knowledge. As one midwife reported,

*“We do not receive any on job training [caring for adolescent mothers] except for what we learn in a school*,* deemed to be not sufficient.” (*MA).

In this similar context, some participants added that since they lack this knowledge and skills, they must wait for specialized providers such as mental health nurses or social workers to provide support. They noted how waiting for providers with specialized skills can impact care provision since they do not have enough or are not always available at the health center. For instance, as one nurse said:

*“We all [healthcare providers] need the training to care for adolescent mothers. There is a time when you receive them and realize you are not trained; it’s an arrangement. We need that training to not wait for those in charge of mental health or with additional training to help adolescent mothers.”* (ND).

A number of health center managers have acknowledged the need for nurses and midwives to receive specific education and training related to the care of adolescent mothers in perinatal services in order for them to feel prepared and confident in this area of practice. For example, one health center manager noted,

*“We can’t say we had a special experience because we didn’t get in-depth training to care adolescent mothers. We got trained a few times*,* and it is not enough.”* (KI**4**).

There is a risk that some nurses and midwives might not be able to respond to the potential effects of trauma and ongoing violence and handle disclosure appropriately due to their lack of knowledge and skills in this domain. It is noteworthy that from the participants’ narratives, the approaches to adolescent mothers vary. In addition, their lack of skills is a potential cause of re-traumatization. For example, as one nurse reported,

*“If you force her [adolescent mother] to talk*,* you can hurt her in one way or another.”* (NG).

In a slightly different vein, a health center manager noted,

*“I have witnessed some cases where nurses and midwives sometimes fail to interact with adolescent mothers because they could not know some trauma signs and symptoms.”* (KI**4**).

(d) ***Physical structure of the perinatal environment***

A few nurses and midwives also highlighted the challenges they face maintaining confidentiality as associated with the services and structure of the clinics. For example, when adolescent mothers leave the health center for the first time, like other mothers, they are given some medical materials such as insecticide-treated bed nets (ITNs) for malaria prevention and other medical supplies. In the community, ITNs from the hospital signals that you are pregnant. As one midwife noted in the following,

“*They are reluctant to take them [ITNs] home*,* saying whoever sees them will think they are pregnant.”* (MD).

Nurses and midwives said they struggle to convince the adolescent mothers that the important thing is the health of their babies and themselves and sometimes they find a way to help assist with this. For example, this midwife will sometimes buy an envelope so to conceal the ITNs. However, teaching about the advantages of ITNs for the health of the adolescent mothers and their babies is a challenge given this reality. The study participants reported that many of these adolescent mothers exhibit a reluctance to sit with other mothers in the common waiting areas of the perinatal services, i.e., ANC. They often approach the nurses and midwives, expressing a desire to speak privately seeking arrangement for not sitting in the common areas with other women who may be their neighbors. This behavior is consistently driven by feelings of stigma related to their pregnancies. To accommodate these concerns, the nurses and midwives typically lead the adolescent mothers to a different entrance, demonstrating a sensitivity to their need for privacy and a supportive approach to their care, even though the environment itself is not set up in a way that supports this. A midwife provided an example related to the perinatal environment in the following quote:

*“I have seen many adolescent mothers coming and not sitting with others. They see me and say hey*,* I want to tell you something. They all had the same issue of wanting to avoid sitting in the waiting area with others. Immediately I led them to another entrance; you saw that we have two doors*,* an entrance and an exit. They usually tell me they are worried*,* ashamed and afraid of what they have experienced.*”(MC).

Some nurses and midwives expressed a need for specialized and sensitive approaches in their practice, especially to support the adolescent mothers’ unique needs. They gave examples of some primary healthcare settings services, where patients are provided with a separate seating arrangement to ensure privacy and reduce stigma. Similarly, they advocated for creating special spaces for adolescent mothers, suggesting that these adolescent mothers should be received in areas with separate entrances and exits. This setup would help maintain their privacy, enhance their comfort, and provide a more supportive environment tailored to their unique needs. As participants suggested:

*“It should be done like in anti-retroviral therapy (ART) services*,* for example. Those who come for medication in the ART service are sitting on their own; they don’t make them sit with others who come for a routine check-up.”* (NE).

*“They [adolescent mothers] should be received in a place where there is an entrance different from the exit because it helps them.”* (MD).

*“These adolescent mothers are ashamed of sharing the waiting areas with others since they do not want them to know what they are doing there. It would be better to have their areas for ANC or even separate exit doors so that no one sees them returning from these services.”* (KI**1**).

#### Theme 4: vicarious trauma

The majority of nurses and midwives interviewed in this study became involved emotionally with adolescent mothers and noted they are often consumed with thoughts about the adolescent that seep into their personal lives. They reported that sometimes they live the feelings of adolescent mothers, become depressed or project the same stories onto their own children, and become hypervigilant in parenting.

- ***Living the feelings***

A few nurses and midwives reported that during the conversations with adolescent mothers, they sometimes feel emotionally exhausted by the contextual features of the adolescents’lives.

The study participants experience significant emotional and psychological challenges in their roles of caring for adolescent mothers. They often feel deeply saddened and shocked by the cases they encounter. The continuous experience of the adolescent mothers’ problems in their minds leaves them feeling helpless and unable to find ways to console them, often leading to feelings of being traumatized. A midwife shared the story of a 17-year-old who went on a trip with her female friends who had also invited boys. The adolescent mother was raped repeatedly after being locked in a room. Thus she did not know the father of her baby. Her family rejected her, and she wandered on the streets. This midwife said,

*“I heard that case*,* and I felt so sad; it touched my heart*,* and I felt shocked.”* (MD).

Another nurse notes,

“*All their [adolescent mothers] problems accumulate in my head*,* and I could not find ways to console them. It’s challenging and traumatizing.”* (NC).

(b) ***‘I carry their Stories Home’***.

Some nurses and midwives said that adolescent mothers’ stories negatively affect them to the extent that they become depressed or project the same stories onto their children. They said that when they are home or outside work, they continue to think about the adolescent mothers’ stories. For example, as one midwife noted,

*“Absolutely! It’s challenging for me because I also have adolescents*,* which affects me. When I leave seeing a case like that*,* I immediately imagine it on mine… So I directly see that it has affected me because I go and take more time to teach mine or even take time to think if it had happened to mine. Sometimes I spend a long time thinking about that. You might be upset or even cry alone.”* (MB).

Carrying these stories outside the perinatal environment, crying alone and projecting them onto their children, signals that caring for adolescent mothers can sometimes be traumatizing for the provider.

(c) ***Hypervigilance in parenting***

Four nurses and midwives reported that they sometimes do not trust males who approach their daughters or close female family members because of what they experience when caring for adolescent mothers. Others do not wish to understand that their daughters are dating. One midwife said that because of caring for adolescent mothers and hearing the stories, for example of being raped by their family’s friends or even by family members, they [the midwives] don’t want their daughters to have relationships with any males. This midwife said,

“*As a parent*,* I am now not happy to see any male who starts these relationships with my daughter. Their [adolescent mothers] family’s friends*,* neighbours and even some family members are the ones who impregnated them.”* (MB).

Another nurse said that when they teach their children or others, they refer to those stories, especially emphasizing that men as perpetrators should be accountable for their actions. As one nurse said sadly (with an angry face),

*“I do not tolerate these problems… I*,* personally when teaching my daughters*,* should emphasize on avoiding men; they are the ones who cause the problems.” (*NG).

As these narratives illustrate the consequences of vicarious trauma can be far reaching – even into the personal lives of the healthcare providers.

Even though nurses and midwives are at risk of vicarious trauma, when we asked their heads of health centers about the available programs to protect their staff from vicarious trauma, they said they do nothing. For example, the KIs in the following said,

*“There’s nothing special based on our administration structure.”* KI**2**.

*“I don’t think we have this service for nurses and midwives. They have their own ways of taking care of themselves.”* KI**1**.

These quotes reflect that there is a lack of support for perinatal healthcare providers who are at risk of developing vicarious trauma.

## Discussion

The aim of this study was to explore the experiences of perinatal nurses and midwives as they work with adolescent mothers in primary healthcare settings in Rwanda to inform the delivery of trauma- and violence-informed care (TVIC). In this study, the main themes were relational practice, individual challenges of caring for adolescent mothers, factors contributing to workarounds, and vicarious trauma. The findings from this study indicate a need to integrate TVIC into perinatal services. This is to assist in making the environment safe and welcoming for adolescent mothers, perinatal nurses, and midwives [13].

### Relational practice

As is evident from the participants’ narratives, midwives and nurses interact with young mothers through engaged relational practice in order to support their well-being. In nursing, relational practice/inquiry implies that nurses have a deep understanding of the healthcare needs of their patients within the context of the complex circumstances under which patients are experiencing healthcare and nurses are providing care [46–48]. A relational practice/inquiry approach helps nurses build a trustworthy therapeutic relationship that provides a comprehensive understanding of the unique features of the client’s circumstances [49]. In perinatal services, this approach is very helpful for recognizing and understanding the reality in which adolescent mothers live and is critical for determining how to tackle their unique challenges. Perinatal nurses [and midwives] can engage in relational practice with mothers by being mother-friendly (treating all mothers the same) regardless of their age and other circumstances [18]. However, due to differing contexts, those who face the greatest structural barriers, i.e., adolescent mothers, face greater risks of violence and trauma, and have fewer options for dealing with these issues [13], highlighting the need to provide more support to adolescent mothers than others.

In our findings, nurses and midwives found it challenging and time-consuming to care for adolescent mothers since most of these mothers are worried, ashamed, shy, distressed and they internalize the stigma when it comes to being pregnant. These findings are consistent with a study conducted in Zambia where midwives reported that caring for adolescent mothers was challenging since it was difficult for them to follow instructions provided by midwives [36]. This explains why, when utilizing a TVIC approach, it is critical to make sure that a clear explanation of the procedures/services to be received and what follows is available or provided [13]. In addition, nurses and midwives should be aware that every behaviour has a reason, e.g., in the context of this study, many of the adolescent mothers had experienced violence – it is essential to be cognizant of the universal nature of violence [13]. Thus, it is important for healthcare personnel to be aware of the challenges associated with caring for adolescent mothers in order to provide safe care.

The findings of the current study underpin the fundamental importance of nurses and midwives being creative and flexible and able to juggle multiple skills to connect with adolescent mothers. In some studies, perinatal nurses were shown to strive to provide a positive experiences to assist adolescent mothers because of the unique issues adolescent mothers present [18, 35]. However, in others, nurses and midwives reported they had negative attitudes toward adolescent mothers [37, 50]. In work by Wilson and colleagues, it was noted that healthcare professionals may have assumptions about adolescent mothers’ behaviours, which can affect how they provide care [51]. Certainly, some adolescent mothers’ behaviors are thought to be associated with the lack of mental preparedness for motherhood [37, 52, 53]. Therefore, it is important to adapt TVIC principles to ensure that healthcare professionals are aware of the potential biases they may have when caring for adolescents. TVIC encourages people to think critically and ask questions to challenge traditional ways of thinking; it also enables them to examine their implicit biases, assumptions, and worldviews and provides a gentle “on-ramp” for developing and sharing new perspectives on service users [13]. In this way, TVIC encourages people to create meaningful dialogue, challenge themselves, and ultimately create positive change.

In this study, nurses and midwives reported that they advocate for adolescent mothers in order to help them pay for some of their healthcare services and find the resources to meet their basic needs. Results similar to these have also been reported elsewhere [54]. Financial hardships can make it difficult for adolescent mothers to afford healthcare services [55–59]. In addition, nurses and midwives demonstrated positive relational practice by adopting interdisciplinary approaches. As an example, they reported involving mental health nurses, social workers, and nutritionists in the care of adolescent mothers. Other research has reported how multi/interdisciplinary teams are essential when caring for adolescent mothers due to their unique needs [51, 60]. When the Ministry of Health is appointing healthcare providers, it is very important to allocate interdisciplinary teams in primary healthcare settings to ensure adolescent mothers receive adequate care. In addition, in this study, and in keeping with findings of a study conducted in Zambia [36], most nurses and midwives emphasized the importance of listening to their adolescent clients with empathy.

### Individual challenges of caring adolescent mothers

Nurses and midwives reported dealing with individual challenges when caring for adolescent mothers in this study such as a lack of knowledge and skills specific to the care of adolescent mothers and the gender of the healthcare provider. Gaps in performing procedures safely when caring for adolescent mothers such as non-pharmacological pain management, family planning, and perineal care were also reported in previous research studies [61, 62]. These gaps can be addressed by educating and training nurses and midwives in adolescent-specific care and providing more resources and support systems to ensure adolescent mothers receive quality care and support. In addition, all male participants in this study reported that adolescents did not allow them to touch them, particularly when they need to conduct vaginal examinations. These findings corroborate the findings of other studies where adolescent mothers preferred female providers [38, 63, 64]. However, in contrast, in a study conducted in South Africa, perinatal women preferred being cared for by males because they were respectful and understanding [65]. For some people, because of cultural norms and religious beliefs, it is not acceptable for a male to care for a woman [66]. The gender of the health care provider and the preferences of adolescent mothers should be considered when assigning healthcare providers within perinatal services. This approach meets with the TVIC principle of fostering opportunities for choice, collaboration and connection [13].

### Factors contributing to workarounds

Workarounds related to inflexible guidelines, lack of protocols and guidelines tailored to adolescent mothers’ needs, limited nurses’ and midwives’ training, and the physical structure of the perinatal environment were also identified as themes in this study. Scholars have coined the term ‘workaround’ as a means by which a professional body or group achieves a specific goal when they are confronted with an obstacle to achieving that goal [67, 68]. Nurses and midwives use workarounds when they lack time or tools to complete their tasks according to guidelines/manuals etc. [69]. In our study, nurses and midwives reported that sometimes rules and regulations prevent them from helping adolescent mothers, such as requiring the father of the baby and proof of community health-based insurance at the first appointment. Despite these rules, they said they would help adolescent mothers. Being challenged by policies and guidelines was also reported in Jamaica by midwives working with adolescent mothers [70]. In the current study, it was mentioned that there are no protocols and guidelines for caring for adolescent mothers and it is difficult to provide tailored care. A study conducted in Namibia also reported the lack of specific guidelines and protocols [59]. In this context, organizations need to develop guidelines and protocols underpinned by the principles of trauma and violence-informed care to assist nurses and midwives in the provision of quality care to adolescent mothers in perinatal services [13].

Perinatal nurses and midwives have reported limited education and training in caring for adolescent mothers. As a result, they are reliant on what they learned in school, which they deemed as insufficient. Limited training for perinatal staff has been reported in Rwanda and other countries [23, 71, 72]. Consequently, and as an example, it is challenging for nurses and midwives to address some of the sexual health aspects relevant to midwifery practice during perinatal care, which negatively impacts the quality of care.

In the current study, nurses and midwives reported that Rwanda’s perinatal environment was not welcoming to adolescent mothers. Midwives and nurses have reported receiving adolescent mothers who do not wish to sit with other mothers due to a sense of not belonging. Adult mothers often stigmatize adolescent mothers when they learn they are pregnant and adolescent mothers themselves can internalize stigma. Nurses and midwives said they tried to find an unconventional way to receive adolescent mothers. They suggested using another service or door to meet their adolescent mothers’ needs, which is a form of workaround. Workarounds introduce hazards, inefficiencies, or errors that affect subsequent steps and activities and may result in noncompliance with management’s intentions [73]. When a workaround involves more complex tasks than the original work process, it becomes less efficient [74].

### Vicarious trauma

In the participants’ narratives, vicarious trauma (VT) was identified as a key finding. According to Kennedy and Booth “VT is a psychological phenomenon that causes a permanent cognitive shift in the inner experience and world views of nurses after prolonged empathetic engagement with a patient’s trauma” [75]^p.1^. In the current study, nurses and midwives shared their experiences of how adolescent mothers’ stories and struggles affected them. As an example, they said they often felt sad listening to them. They report that they live with their feelings, carry their stories out of the workplace and find themselves hypervigilant and perhaps overprotective with regards to parenting their daughters. A recent rapid evidence assessment found that working with clients who experienced sexual violence has several negative impacts, including developing trauma symptoms, social relationship disruptions, changes in individual behaviors, and psychological and emotional distress [76]. As such, providers may face unsafe conditions in the perinatal environment, which calls for interventions that make the environment safe so providers can provide quality care.

### Strengths and limitations

This study possesses strengths and limitations that must be considered when interpreting the findings. In terms of strengths, the research employed an ID qualitative approach, which yields a comprehensive and in-depth understanding of clinical problems in nursing practice. This qualitative approach allows for generating practical solutions that can be effectively applied in real-world settings [26]. Additionally, the study employed triangulation to enhance our understanding of nurses’ and midwives’ experiences caring for adolescent mothers. In this study we collected information from multiple perspectives to provide a comprehensive view of nurses’ and midwives’ experiences.

However, it is pertinent to acknowledge this study’s limitations. Firstly, the participants were sourced from eight primary healthcare settings in Rwanda. Due to the cultural specificity of the setting, the findings presented in this study may not be universally applicable in diverse cultural or regional settings. Factors such as variations in healthcare systems, cultural norms, and available resources may significantly impact the applicability of the results elsewhere. We comprehensively documented and contextualized the cultural and systemic factors that could influence our findings, providing readers with a detailed background and methodology. To enhance the credibility and transferability of the results, we employed triangulation methods, utilizing multiple data sources and cross-referencing with existing literature.

Second, we acknowledge the potential presence of social desirability bias within the study. Some participants might have over-reported, under-reported, or even failed to report certain practices [77]. To address this, we ensured that participants remained anonymous and their responses confidential, fostering a safe space to honest expression. We employed indirect questioning techniques, enabling the study participants to express their attitudes and practices in a more indirect and non-judgmental way. Additionally, we utilized trained and neutral RAs to reduce interviewer bias. We also applied triangulation of data sources to cross-check the information obtained. Lastly, we used interview questions which encouraged the study participants to share specific examples or experiences of their practices in perinatal services, minimizing the likelihood of socially desirable answers.

Third, since we selected experienced participants who were willing to share their experiences, we acknowledge the selection bias and it is possible that certain participants with different perspectives were overlooked. To address this in our study, we conducted a thorough screening process to ensure the inclusion of the study participants with diverse experiences related to our research question. Additionally, we employed triangulation to capture a variety of perspectives from nurses and midwives and their managers and minimize relying on a single source of information. Lastly, we regularly held peer debriefing meetings and remained open to feedback throughout the study. Fourth, Thorne and colleagues [78] note that the ID does not produce the new truth; rather, it generates what they considered “tentative truth claim” about shared clinical phenomenon. Thus, we acknowledge that further analysis and future studies can generate different findings.

Future research should aim to enhance incorporate a more diverse range of participants from various primary healthcare settings located in different regions and cultural backgrounds. This approach will foster a more comprehensive understanding of the experiences of nurses and midwives providing care to adolescent mothers in various contexts. Moreover, adopting a mixed-methods approach could further enrich the data by merging the depth of qualitative insights with the breadth of quantitative analysis. Secondly, conducting longitudinal studies is essential to understand how nurses’ and midwives’ experiences evolve over time and in response to changes in healthcare policies and practices. This approach offers a dynamic perspective on the challenges faced and strategies employed when caring for adolescent mothers. These insights can inform long-term interventions. Lastly, future research should focus on minimizing social desirability and selection biases.

## Conclusion

Nurses and midwives encounter various challenges in caring for adolescent mothers; their unique requirements necessitate creativity, flexibility, and attentive listening. Nurses and midwives encounter specific challenges, including a lack of special knowledge regarding adolescent mothers. In addition, they face limitations imposed by inflexible guidelines and a lack of protocols and procedures. Furthermore, the perinatal environment where nurses and midwives work may not be conducive to ensuring adolescent mothers’ safety and well-being. Additionally, many nurses and midwives experience vicarious trauma as a significant practice challenge, which can affect their mental health and well-being. Therefore, perinatal guidelines and protocols are needed specifically for caring for adolescent mothers in these contexts. TVIC training and ongoing professional education should be provided to increase nurses’ and midwives’ knowledge of caring for adolescent mothers, preventing re-traumatization and mitigating vicarious trauma. Finally, organizations should develop strategies and avail resources to support nurses and midwives at risk of and to prevent vicarious trauma.

### Electronic supplementary material

Below is the link to the electronic supplementary material.

Supplementary Material 1

Supplementary Material 2

## Data Availability

The datasets used and/or analyzed during the current study are available from the corresponding author on reasonable request.
