# Supplementary material for: “… I carry their stories home …”: experiences of nurses and midwives caring for perinatal adolescent mothers in primary health care settings in Rwanda
Source: BMC Nurs. 2024 Sep 2;23:609. doi: 10.1186/s12912-024-02247-7 (PMC11368027; doi:10.1186/s12912-024-02247-7)
Supplement: Supplementary file 1 — Supplementary Material 1 [file 12912_2024_2247_MOESM1_ESM.docx]

**Interview guide- Nurses and midwives**

The interview will last approximately 30-60 minutes. Interview will be audio-recorded. I assure you that your discussion will be confidential and no name or other socio-demographic data will be pronounced in this interview. I will use open ended questions and you will not be asked questions that you have previously answered. If you feel uncomfortable to answer a question, let me know I will skip it and there will be no negative impact on your participation. Please feel free to ask any question before we continue. Thank you very much.

1. Tell me what brought you to participate in this discussion today?
2. Tell me what brings adolescent mothers to your particular service – what do you provide? What are the unique features of care for adolescent mothers?
3. As you know, not all, but many adolescent mothers have experienced sexual violence. How do you ensure adolescent mothers experience safety and comfort in this setting?
4. Tell me about the philosophy of care provision as it pertains to adolescent mothers and perinatal services.
5. What are the outcomes you hope for in working with adolescent mothers?
6. What are the highlights of your work with adolescent mothers?
7. What are the strengths related to care provision with adolescent mothers in this setting?
8. What are the challenges as you see them in providing care to adolescent mothers in this context?
9. Can you tell me about your overall experience caring for adolescent mothers in perinatal services? Perhaps you could provide a couple of examples of what care you have provided.
10. What was your educational preparation in the care of people who have a violence history? What do you see as your need in this regard?
11. What are your recommendations moving forward to improve the care of adolescent mothers in perinatal services?
12. Is there anything else can you share with me that you think is important for this research to know how perinatal services are offered to adolescent mothers, that we did not cover in this discussion?
