# Supplementary material for: “… I carry their stories home …”: experiences of nurses and midwives caring for perinatal adolescent mothers in primary health care settings in Rwanda
Source: BMC Nurs. 2024 Sep 2;23:609. doi: 10.1186/s12912-024-02247-7 (PMC11368027; doi:10.1186/s12912-024-02247-7)
Supplement: Supplementary file 2 — Supplementary Material 2 [file 12912_2024_2247_MOESM2_ESM.docx]

**Invitational questions (key informants- Heads of health centers)**

The interview will last approximately 30-60 minutes. Interview will be audio-recorded. I assure you that your discussion will be confidential and no name or other socio-demographic data will be pronounced in this interview. I will use open ended questions and you will not be asked questions that you have previously answered. If you feel uncomfortable to answer a question, let me know I will skip it and there will be no negative impact on your participation. Please feel free to ask any question before we continue. Thank you very much.

1. Can you tell me what interested you in my research study?
2. Tell me what it is like overseeing perinatal services as a head of health center?
3. Please share with me your experiences of how perinatal services are provided to adolescent mothers in this institution
4. How do perinatal services’ practices, policies, and procedures include a focus on trauma and violence, and issues of safety and confidentiality for adolescent mothers in your institution?
5. In what ways do you offer support to nurses and midwives to provide the perinatal services to adolescent mothers?
6. What helps you in creating an environment supportive of nurses and midwives to provide perinatal services to adolescent mothers in this institution?
7. What do you see as barriers to you in creating an environment supportive of nurses and midwives to provide perinatal services to adolescent mothers in this institution?
8. In what ways do you involve clients, nurses, and midwives in identifying ways to implement perinatal services?
9. How do perinatal services’ policies, practices, and procedures foster opportunities for choice and connection for adolescent mothers?
10. How are the perinatal services’ policies, practices, and procedures tailored to adolescent mothers’ needs, strengths, and contexts?
11. What do you think can be done to improve perinatal services of adolescent mothers in this institution?
12. Is there anything else can you share with me that you think is important for this research to know about the current organizational culture of health center/health post and how it influences how perinatal services are offered to adolescent mothers, that we did not cover in this discussion?
